# Case report: Treatment of persistent atypical odontalgia with attention deficit hyperactivity disorder and autism spectrum disorder with risperidone and atomoxetine

**DOI:** 10.3389/fpain.2022.926946

**Published:** 2022-07-22

**Authors:** Satoshi Kasahara, Chihiro Takao, Ko Matsudaira, Naoko Sato, Trang Thi Huyen Tu, Shin-Ichi Niwa, Kanji Uchida, Akira Toyofuku

**Affiliations:** ^1^Department of Anesthesiology and Pain Relief Center, The University of Tokyo Hospital, Tokyo, Japan; ^2^Department of Pain Medicine, Fukushima Medical University School of Medicine, Fukushima, Japan; ^3^Department of Psychosomatic Dentistry, Graduate School of Medical and Dental Sciences, Tokyo Medical and Dental University, Tokyo, Japan; ^4^Department of Medical Research and Management for Musculoskeletal Pain, 22nd Century Medical and Research Center, The University of Tokyo Hospital, Tokyo, Japan; ^5^Nursing Department, The University of Tokyo Hospital, Tokyo, Japan; ^6^Department of Basic Dental Science, Faculty of Odonto-Stomatology, The University of Medicine and Pharmacy at Ho Chi Minh City, Ho Chi Minh, Vietnam; ^7^Department of Psychiatry, Aizu Medical Center, Fukushima Medical University, Aizuwakamatsu, Japan

**Keywords:** attention deficit hyperactivity disorder, autism spectrum disorder, atypical odontalgia, chronic primary pain, risperidone, atomoxetine, anger, irritability

## Abstract

Chronic pain has recently been associated with developmental disorders [autism spectrum disorder (ASD) and attention deficit hyperactivity disorder (ADHD)]. Regarding chronic pain in adulthood, fibromyalgia, migraine, and chronic low back pain have been associated with ADHD. The ICD-11 disease classification categorizes these pain diseases as chronic primary pain, suggesting high comorbidity with developmental disorders in chronic primary pain. Atypical odontalgia (AO) is a persistent tooth pain that occurs in the absence of any of the usual dental causes, most of which are triggered by dental treatment. Conditions characterized by tooth pain with no apparent cause are also classified as chronic primary pain. Approximately half the patients with AO are diagnosed with psychiatric disorders; the most common are depression (15.4%) and anxiety disorders (10.1%). However, there are no reports on neurodevelopmental disorders comorbid with AO. In the present study, we report a case of a 46-year-old man with numerous complaints (e.g., occlusal instability, difficulty eating, difficulty speaking), who took work leave due to worsening of his symptoms after periodontal scaling (“gingival recession” and “aggressive periodontal treatment”) and frequently expressed dissatisfaction and anger at the hospital, making the dental treatment difficult. After a referral to a psychiatrist specializing in chronic pain, AO and previously undiagnosed comorbidity of ASD and ADHD were confirmed. Atypical antipsychotic risperidone for ASD irritability and an ADHD medication, atomoxetine dramatically reduced anger, pain, anxiety, depression, and pain catastrophizing thoughts, leading to reduced obsession with his symptoms and less frequent complaints. After risperidone (1 mg/day) + atomoxetine (120 mg/day) were ultimately prescribed after adjustment, he was able to return to work 226 days after initiation of psychiatric treatment. Recent studies show that comorbidity of developmental disorders in patients with chronic pain is likely to be undetected. Clinicians should include screening for ASD and ADHD not only in cases of fibromyalgia, migraine, and chronic low back pain, but also in orofacial pain such as AO and other treatments for chronic primary pain. For patients diagnosed with ASD or ADHD, an effective drug therapy for ASD and ADHD should be considered.

## Introduction

Atypical Odontalgia (AO) is a persistent tooth pain that occurs in the absence of any of the usual dental causes, most of which are triggered by dental treatment (1).

It has been reported that approximately half (48–68%) of AO patients have comorbid psychiatric disorders (2, 3). In the study with the largest number of subjects (*N* = 383), 46% of AO patients had comorbid psychiatric disorders, most commonly depression (15.4%) and anxiety disorders (10.1%), while more serious psychoses such as schizophrenia (1.8%) and bipolar disorder (3.0%) were rare (1). However, there are no reports on neurodevelopmental disorders comorbid with AO. Dental treatments may become a trigger for chronic oral pain, and dental staff lacking insights into these conditions may be confused by unexpected patient reactions.

Attention deficit hyperactivity disorder (ADHD) and autism spectrum disorder (ASD) are the most frequently recognized neurodevelopmental disorders. ADHD is characterized by developmentally inappropriate inattention, impulsivity, and/or hyperactivity, which causes disability in relatively long-term and across multiple domains of life activities. ASD is characterized by persistent deficits in social interaction and communication, as well as restrictive, repetitive patterns of behavior or interests (4).

Regarding the association between chronic pain and adult developmental disorders, the only reports on ASD have been for children and adolescents (5, 6), but there have been reports on ADHD and its association with fibromyalgia, migraine headaches, and chronic low back pain. Patients with fibromyalgia have been reported to have a high frequency (25–80%) of complications with ADHD (7–13). In patients with FM with ADHD, ADHD treatments such as psychostimulants have also been reported to improve both ADHD and pain (9). In addition, a meta-analysis study showed an association between ADHD and migraine (14). Regarding chronic low back pain, it has been reported that 31.7% of patients with chronic low back pain have clinical ADHD symptoms (15). It has also been reported that ADHD is associated with central sensitization in patients with chronic low back pain (16). These fibromyalgia, migraine, and chronic low back pain are classified as chronic primary pain in the disease classification of ICD-11 (17), suggesting high comorbidity of ADHD in patients with chronic primary pain. Chronic orofacial pain, including AO, is also classified as the chronic primary pain. However, no study has reported an association between AO and developmental disorders.

In many cases, AO patients do not respond to conventional drug therapy, given the high comorbidity of mental illness. Therefore, the discovery of a new medication for the treatment of AO is needed (18, 19). Atypical antipsychotics have recently drawn attention as an approach corresponding to pathological hypothesis (2) that persistent idiopathic facial pain, such as AO, is caused by dysfunctional brain dopamine activity; however, only aripiprazole has been investigated (20).

In this study, we report a case in which atypical antipsychotic risperidone and atomoxetine dramatically reduced anger and pain in a patient with adulthood AO comorbid with ASD and ADHD.

## Case description

Mr. A was a 46-year-old single, male.

### Chief complaint

Chronic pain in the left maxillary and right mandibular molar regions (according to him, due to gingival recession caused by periodontal treatment), occlusal discomfort (specific teeth unidentified), extracted teeth, teeth sticking to the lips, occlusal instability, difficulty in opening the mouth, eating, and speaking.

### Medical history

None.

### Present illness

He began visiting a nearby dental clinic in 2018. On May 7, 2019, he was referred to the clinic of periodontics at the Tokyo Medical and Dental University dental hospital by the nearby dentist due to problems with oral hygiene instructions owing to complaints such as “gingival damage due to tooth brushing” and many other complaints (e.g., occlusal instability, difficulty eating, difficulty speaking). At the clinic, he mentioned that his teeth were sensitive to cold water and did not understand the explanation of the lack of identifiable causes requiring treatment. Thereafter, he had repeated unscheduled visits and phone consultations with the clinic to present with complaints such as “I feel some teeth are missing,” “I can no longer chew with the left teeth.” In July, periodontal scaling was initiated. However, his complaints increased and worsened (e.g., “gingival recession,” a tingling sensation and numbness,” and “the lower teeth are damaged by the upper teeth”). He began visiting the clinic approximately once or twice a week. During 3 months after August, he lost 20 kgs due to “hip pain” and “difficulty walking.” From October, he was unable to work and took leave. The Department of Psychiatry, to which he was recommended by his younger sister, suspected he had somatic symptom disorder after excluding psychotic disorders, depression, and bipolar disorder. However, the psychiatric treatment was discontinued because he remained highly dissatisfied with the dental treatment.

**Figure 1:**
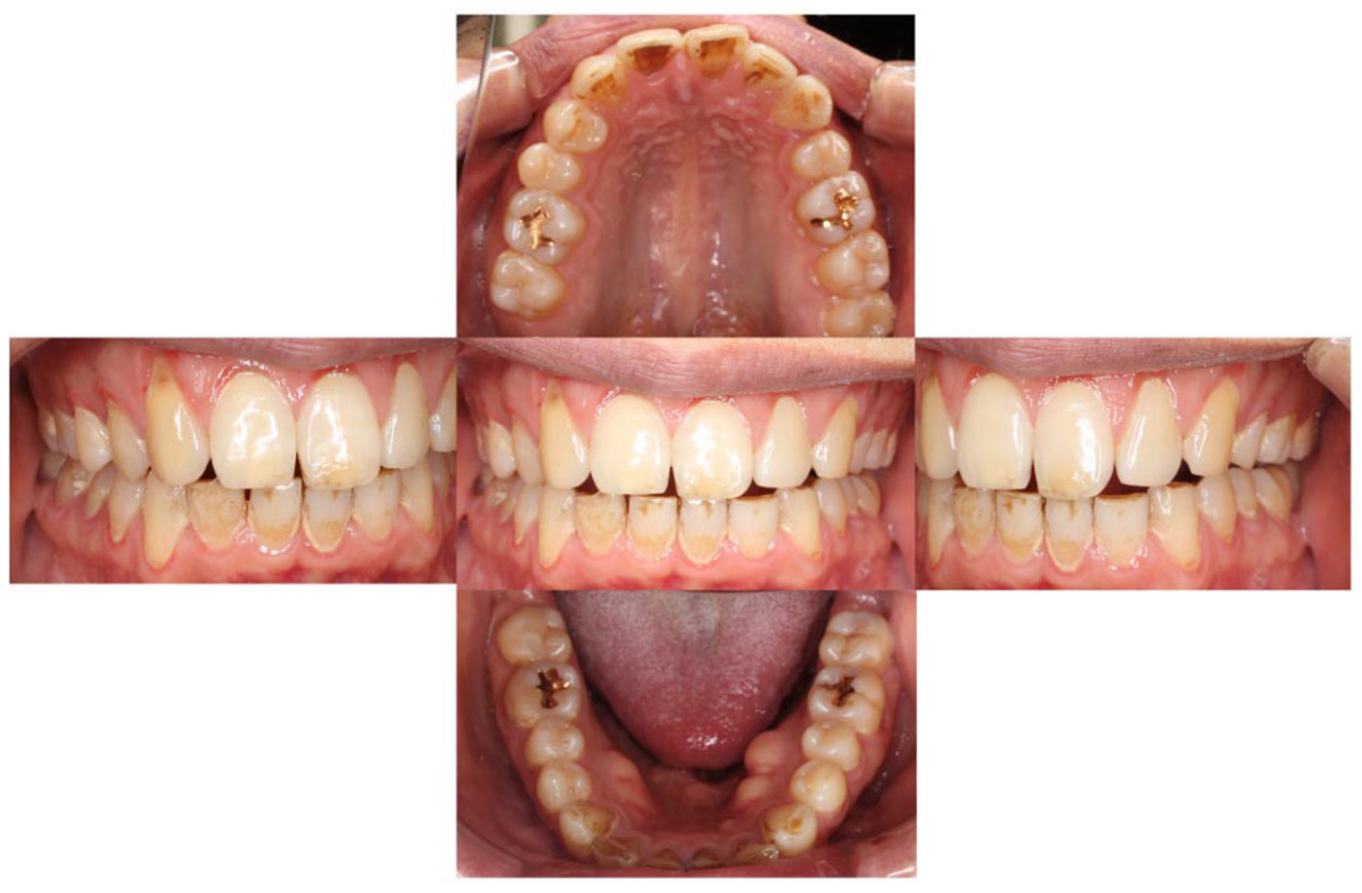
Intra-oral findings at patient's first visit to psychosomatic dentistry clinic.

On November 26, 2019, he visited our clinic (Department of Psychosomatic Dentistry) for the first time. However, the examination did not reveal any findings correlated with his complaints (Figure 1). Mr. A was diagnosed with AO, regarding that his pain was limited around his teeth (however, moving beyond nerve distribution), not provoked by jaw movement and failed to respond to every conventional dental treatment. However, based on the previous treatment process and his attitude, underlying developmental disorders were suspected. Hence, effects of amitriptyline and so on were not anticipated. He was referred to the outpatient department of SK, who is familiar with the treatment of developmental disorders in the Department of Anesthesiology, The University of Tokyo Hospital; at this point, the patient declined the referral.

**Figure 2:**
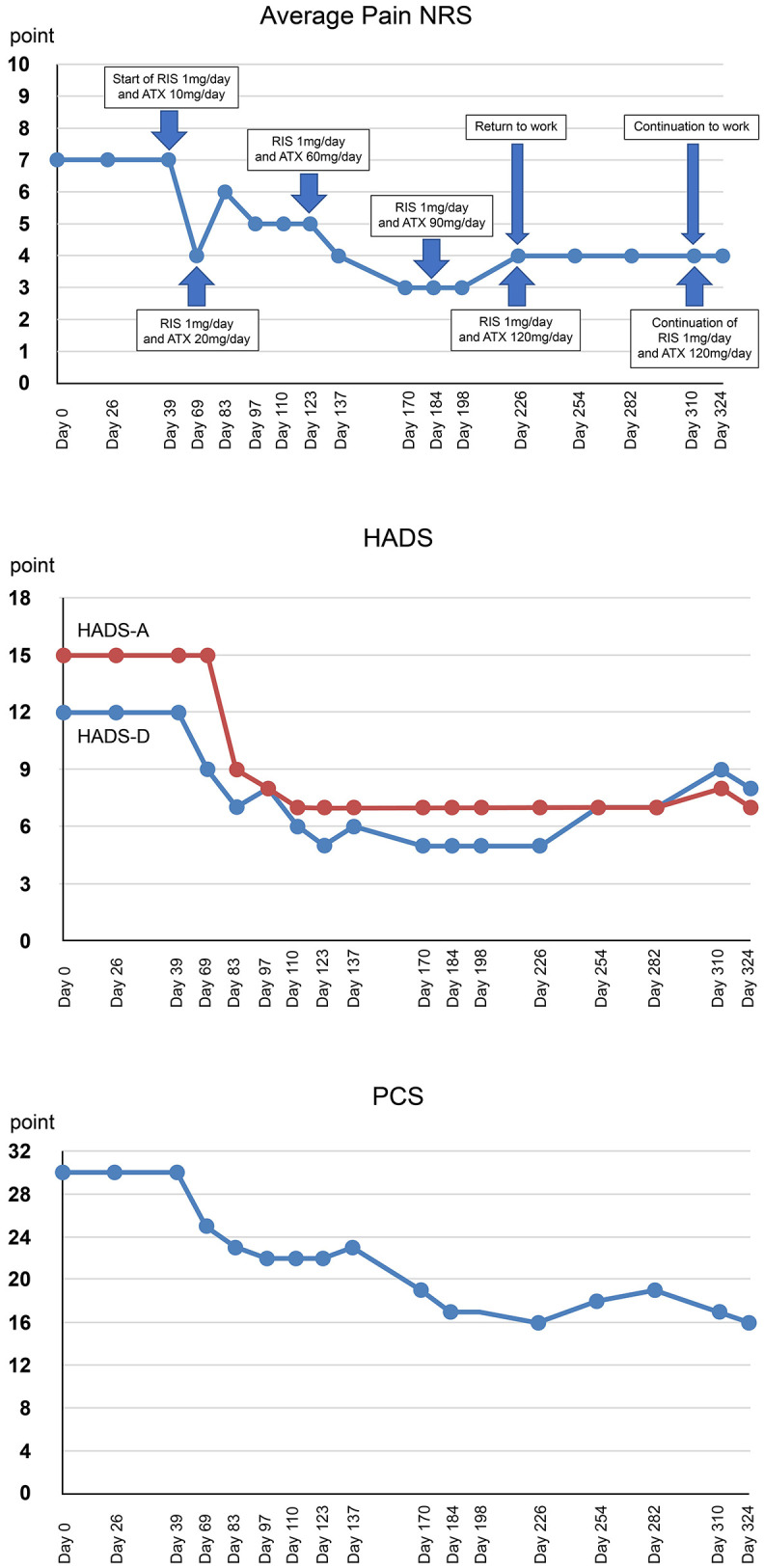
Timeline of events and medication and changes in the rating scale over the course of treatment. RIS, risperidone; ATX, atomoxetine; NRS, numerical rating scales; HADS-A/D, hospital anxiety and depression scale-anxiety/depression; PCS, pain catastrophizing scale.

However, given his continued complaint calls and letters regarding “incidents at the periodontics clinic” to the chief of periodontics clinic, our dental hospital director issued a treatment refusal document and send it to the patient in April. In response to our letter, the patient made repeated visits and phone consultations with our department, asking “What should I do with my teeth?” Therefore, he was advised to seek treatment at the University of Tokyo Hospital. Despite reluctance, on March 6, he eventually agreed to the referral. He was referred to Dr. SK a psychiatrist at the Department of Anesthesiology and Pain Relief Center, on April 24, 2020 (day 0). The timeline for this patient is shown in Figure 2. During the examination, he was suspicious of the treatment in the primary referral hospital and made unsolicited complaints without listening to the advice of Dr. SK. A structured interview, the Mini-International Neuropsychiatric Interview (21), was conducted to differentiate comorbid psychiatric disorders. He did not have a major depressive episode with hypochondriac delusions or other melancholy-type features, a manic episode, or psychotic disorder. He displayed gender-atypical gestures and abnormal eye contact and ritualized patterns of verbal or non-verbal behavior (his speech was excessively formal and polite). He was overly particular about Dr. SK's wording and frequently corrected his expressions. He had highly restricted and fixated interests in gingival pain and was hyperreactive to sensory input. Hence, he was diagnosed with ASD according to the diagnostic criteria of the DSM-5 (4). He was highly educated and employed in a highly structured, professional occupation, and did not have severe impairment in social functioning due to ASD prior to the onset of AO.

Based on hyperactivity and impulsivity symptoms observed (fidgeting with hands and squirms in seat, blurting out answers, interrupts others), the long version of the Conners' Adult ADHD Rating Scale (CAARS-S) and the observer-rated (CAARS-O) questionnaire (22) were administered to examine the suspected comorbidity of ADHD. Mr A's percentiles of the subscale of the CAARS-S were: E. DSM-IV Inattentive Symptoms, 90; F. DSM-IV Hyperactive-Impulsive Symptoms, 98; G. DSM-IV ADHD Symptoms Total, 96; H. ADHD Index, 88. According to ADHD symptoms in the CAARS, Mr. A's ADHD symptoms were at the clinical level of psychiatry (Table 1). In addition, according to the CAARS-O completed by his younger sister, the percentile of the subscale D. Problems with Self Concept was 99, indicating low self-esteem related to ADHD symptoms. His developmental and life history was obtained and evaluated with the CAARS using the Diagnostic Interview for Adult ADHD (23). The results indicated inattention: poor listening to others since childhood; avoiding tasks requiring sustained mental effort, poor logical thinking; easily distracted by ambient noise; forgetful in daily activities. Accordingly, he was diagnosed with ADHD, predominantly inattentive type in the DSM-5 (day 26).

**Table 1:** The patient's score on CAARS-S (in percentile rank).

**Treatment stage**	**CAARS**
Diagnostic stage, before ADHD-specific treatment.	DSM-I = 90 DSM-Hy/I = 98 DSM-Total = 96 ADHD-Index = 88
After drug adjustment (RIS 1 mg/day + ATX 120 mg/day)	DSM-I = 60 DSM-Hy/I = 21 DSM-Total = 36 ADHD-Index = 26

*ADHD, attention deficit hyperactivity disorder; CAARS, Conners' adult ADHD rating scales; DSM-I, DSM-IV inattentive symptoms; DSM-Hy/I, DSM-IV hyperactive–impulsive symptoms; DSM-Total, DSM-IV ADHD symptoms total; RIS, risperidone; ATX, atomoxetine*.

During each examination, the pain numerical rating scale (NRS) (24), the Hospital Anxiety and Depression Scale (HADS) (25), and Pain Catastrophizing Scale (PCS) (26) were administered. Subjective pain intensity was assessed using the numerical rating scale (NRS) (24). Regarding the changes in NRS scores, the minimum clinically important difference is called MCID, decreases of−2 points (or−33.0%) or more in NRS is considered to be substantial or optimal (27). The average pain NRS score of Mr. A was high (7 points).

Symptoms of anxiety and depression were assessed using the Hospital Anxiety and Depression Scale (HADS) (25). Based on previous studies, the MCID of HADS is set at 1.5 (28). Mr. A's HADS results revealed clinical anxiety and depression symptoms (HADS-A, 15 points; HADS-D, 12 points).

Catastrophic thinking related to pain was evaluated using the Pain Catastrophizing Scale (PCS) (26). It has been reported that strong catastrophizing thoughts leads to increased pain and disability possibility, resulting in chronic pain (29). The MCID of PCS can be improved by 38–44% (30). Mr. A's PCS results (PCS score 30 points) indicated clinically catastrophic thinking. Changes in pain NRS, HADS, and PCS during treatment course are shown in Figure 2.

Mr. A exhibited strong anger and suspicion at the periodontist who performed scaling. Dr. SK identified the uncontrollable temper outbursts and aggression as ASD irritability and prescribed risperidone (1 mg/day) (day 39). In addition, atomoxetine was initiated at 10 mg to treat severe anxiety and fear due to hyperarousal of ADHD that may contribute to irritability. At the subsequent examination (day 69), the patient responded to medication and stated that “pain was reduced.” Temper outbursts and frequent suspicions and complaints of scaling in the primary referral hospital were reduced and Mr A was more polite and considerate. To prevent discontinuation of medication due to side effects, atomoxetine was carefully increased to 20 mg.

During the examination 2 weeks later (day 83), he was calmer, and his facial expression was less grim, and the amount complaints regarding the symptoms he faced had reduced. Therefore, atomoxetine was increased to 30 mg. During the examination 2 weeks later (day 97) he did not mention anger or suspicion of previous treatments except one mention of oral discomfort. He requested consultation on more practical topics such as the preparation of the document for sickness and injury allowance. Therefore, atomoxetine was increased to 40 mg. During the examination 2 weeks later (day 110), pain and numbness were almost resolved. He stated, “Lately, I have been very stable mentally.” Additionally, he wanted to discuss with his boss when he would return to work, so the doctor created a medical certificate permitting him to return to work. Thereafter, atomoxetine was increased by 10 mg at ~2-week intervals, leading to reduced pain and numbness and improved mental health status. He returned to work after the administration of the maximum dose of atomoxetine (120 mg/day) (day 226) with no side effects indicating discontinuation. He stated, “I am able to set up my work better than before because of the medication I have been taking.” Re-administration of the CAARS-S at day 324 showed the following percentiles for the subscale of the CAARS-S and improved ADHD symptoms: E. DSM-IV Inattentive Symptoms, 60; F. DSM-IV Hyperactive Impulsive Symptoms, 21; G. DSM-IV ADHD Symptoms Total, 36; and H. ADHD Index, 26 (Table 1). Pain NRS (4 points) improved by 3 points, HADS-A (7 points) improved by 8 points, HADS-D (8 points) improved by 4 points, and PCS (16 points) improved by 14 points (46.7%) from the first visit. Clinically significant improvement was observed exceeding MCID on all scales. Thereafter, he continued to visit the clinic once a month to receive medication. Regardless of the presence of mild pain, he continued his work and achieved social integration. He stated that “the pain has improved, and I am now able to do my hobby of reading, which I had to interrupt because of the pain.”

## Discussion and conclusions

This case demonstrated comorbidity of ASD and ADHD in a patient with AO indicating that atypical antipsychotic risperidone for ASD irritability and ADHD drug atomoxetine for ADHD reduce anger and improve persistent AO.

These results show the possibility of comorbidity of ASD and ADHD not only in persistent chronic primary pain (17) (fibromyalgia, migraine, and chronic low back pain) but also in AO. There is no research on chronic pain that examined both ASD and ADHD in adulthood.

According to a pediatric study (5), 38 (26.0%) of 146 pediatric patients aged 8 to 17 years, who visited a tertiary care pain clinic due to chronic pain, were diagnosed with developmental disorders (ASD and/or ADHD), but only 7 (4.8%) were diagnosed with developmental disorders before visiting a pain clinic, indicating that developmental disorders in children with chronic pain are likely to be undetected. Additionally, >80% of adult ADHD is underdiagnosed, even in clinical psychiatry settings (31), suggesting that ADHD in adulthood is also likely to be undetected. Furthermore, ADHD is very often underdiagnosed in the clinical setting of chronic pain, as it is the orthopedic surgeon, pain clinician, or dentist, who is unfamiliar with ADHD practice, sees patients with chronic pain. It has also been reported that 35.6% of patients with developmental disorder who experienced chronic pain in adulthood had both ADHD and ASD (6).

In other words, as developmental disorders are easily overlooked, and it is necessary to confirm both ASD and ADHD in the detailed examination of developmental disorders that coexist with chronic pain in adults. In this case as well, although the patient had clinical levels of ASD and ADHD, there was no history of diagnosis of developmental disorders, but detailed examination using an assessment tool confirmed the coexistence of ASD and ADHD. This led to the prescription of risperidone for ASD irritability and atomoxetine for ADHD, resulting in a significant reduction in anger and pain.

Inattention-dominant ADHD is particularly hypersensitive to their environmental stimuli and has difficulty filtering out noxious stimuli. Thus, patients with this subtype cannot tolerate even mild contact stimuli. In adulthood, they overreact to stressful environments, and their cognitive inability to filter out ubiquitous physical stimuli (such as mild muscle pain or tinnitus) makes them susceptible to chronic pain (32).

ADHD drug atomoxetine is a selective noradrenaline reuptake inhibitor (NRI) that inhibits the norepinephrine transporter in the prefrontal cortex and improves ADHD symptoms by increasing both noradrenaline and dopamine (33). On the other hand, excessive phasic firings of noradrenaline and dopamine due to prolonged severe stress increase anxiety due to severe hyperarousal and worsen ADHD symptoms in ADHD patients too. In such cases, the NRI atomoxetine can desensitize postsynaptic noradrenaline and dopamine receptors and gradually normalize the sustained firing (34). Theoretically, prefrontal cortex performance displays an inverted U curve and is highest in the presence of moderate dopamine and noradrenaline neurotransmission (35). By moderating both neurotransmissions, atomoxetine may have enhanced the performance of the prefrontal cortex to act as a virtual filter, reducing unpleasant stimuli. Hence, atomoxetine has been reported to improve chronic pain comorbid with ADHD (36).

The patient in this study also had anxiety and severe pain that may have been caused by prolonged stress and hyperarousal due to concerns about aggressive gingival scraping. However, it was inferred that atomoxetine normalized excessive neurotransmission and reduced anxiety and pain. The final dose of ATX in this case was 120 mg/day, which was at the acceptable upper end of the adult ATX dosage range (37). Pediatric study has shown that atomoxetine for ADHD with comorbid ASD produced gastrointestinal side effects in most cases (38), but no significant side effects were observed in this case.

Risperidone is an atypical antipsychotic that acts mainly as an antagonist at dopamine D2, serotonin 5-HT2, and noradrenergic α2 receptors (34). The Food and Drug Administration (FDA) approved risperidone as a treatment for irritability in ASD, including tantrum, aggression, and self-injury, in 2006 (39). This is because higher levels of dopamine and/or serotonin are associated with a happier mood, but their excessive neurotransmission is thought to cause psychotic, manic or aggressive behavior (34).

Furthermore, anger has been associated with chronic pain, and reducing anger can contribute to improving pain (40). So, risperidone has been reported to improve chronic pain in angry patients, resulting in higher levels of psychosocial functioning (41). Regarding the dosage of risperidone for irritability in adults with ASD, a mean of 2.9 mg/day was reported to be significantly better than placebo (42), but the 1 mg/day dosage in this case was lower than that. That study also reported that the group receiving an average of 2.9 mg/day of risperidone experienced temporary mild sedation and small weight gain, but with less frequent adverse effects than those reported for children (42). In summary, risperidone may be a treatment option for anger and tantrums in chronic pain comorbid with ASD.

The patient in this study also had strong temper issues and anger regarding the scaling performed at the primary referral hospital, possibly due to ASD irritability. Quick improvement in ASD irritability after treatment with risperidone led to reduced pain, anxiety, and depression, and improved catastrophizing thoughts and frequent problem behaviors in the primary referral hospital, facilitating the dose adjustment of atomoxetine with longer time to efficacy onset.

There is currently no study showing the comorbidity of ASD and ADHD in patients with AO and the positive effects of atypical antipsychotic risperidone for ASD and ADHD drug atomoxetine on anger and AO. Therefore, the patient in this study is the first case with such improved conditions. However, this study has some limitations. First, because of their side effects on metabolic functions such as weight gain, abnormal lipid metabolism, and hyperglycemia even at lower doses than FDA-approved indications, clinicians are recommended to not prescribe atypical antipsychotics as first-line treatment for chronic pain (43). Risperidone should be initiated only when there is no other available standard treatment. Second, because the patient in this study was simultaneously treated with risperidone and atomoxetine, it is unknown which drug contributed to the improvement in anger and/or pain to what extent. Third, although the pharmacological intervention in this case had the advantage of leading to relatively rapid improvement in pain and anger, treatment for developmental disorders should be administered in parallel with pharmacotherapy and psychosocial treatment (44), which limits the generalizability of the interventions in this study.

In this study, we demonstrated that risperidone for ASD irritability and atomoxetine for ADHD can reduce anger and persistent AO. This is a case report of a patient with persistent AO and comorbidity of ASD and ADHD. As described above, chronic primary pain, such as fibromyalgia, migraine, and chronic low back pain, has also been associated with developmental disorders such as ASD and ADHD. Chronic primary pain has been associated with severe emotional distress (e.g., anxiety, anger/frustration, or depression), reduced activities of daily living, and mental disorders (e.g., deficits in social communication) (17). Therefore, neurocognitive characteristics due to developmental disorders such as ASD and ADHD may be involved in the development of emotional distress and mental impairment. Furthermore, idiopathic orofacial pain conditions, such as AO, burning mouth syndrome, and idiopathic facial pain have many characteristics in common. Therefore, it is considered that regardless of the difference in the severity, these three diseases are on the same neuroplasticity pain spectrum developed through dysfunctional brain dopamine activity (2, 45). Future treatment may include the screening of ASD and ADHD for patients with not only fibromyalgia, migraine, and chronic low back pain, but also orofacial pain, such as AO and other chronic primary pain. For patients diagnosed with ASD or ADHD, targeted drug therapy may be prescribed. However, this is the only case report on the association between AO and developmental disorders. Future studies are needed to examine this association further.

## Data availability statement

The original contributions presented in the study are included in the article/supplementary material, further inquiries can be directed to the corresponding author.

## Ethics statement

The studies involving human participants were reviewed and approved by Research Ethics Committees of Tokyo University Hospital and Tokyo Medical and Dental University Hospital. The patients/participants provided their written informed consent to participate in this study. Written informed consent was obtained from the individual(s) for the publication of any potentially identifiable images or data included in this article.

## Author contributions

SK, S-IN, KM, and AT conceived the work and interpreted the data. SK and NS collected the data. SK managed the data. SK, CT, TT, and AT drafted the manuscript. SK, KU, and AT prepared the final manuscript. All the authors approved the final manuscript.

## Funding

This work was supported by JSPS KAKENHI (Grant Numbers JP20K07755 and JP22K10141).

## Conflict of interest

The authors declare that the research was conducted in the absence of any commercial or financial relationships that could be construed as a potential conflict of interest.

## Publisher's note

All claims expressed in this article are solely those of the authors and do not necessarily represent those of their affiliated organizations, or those of the publisher, the editors and the reviewers. Any product that may be evaluated in this article, or claim that may be made by its manufacturer, is not guaranteed or endorsed by the publisher.
